# Supplementary figures and images for: Characterising the mechanisms underlying genetic resistance to amoebic gill disease in Atlantic salmon using RNA sequencing
Source: BMC Genomics. 2020 Mar 30;21:271. doi: 10.1186/s12864-020-6694-x (PMC7106639; doi:10.1186/s12864-020-6694-x)

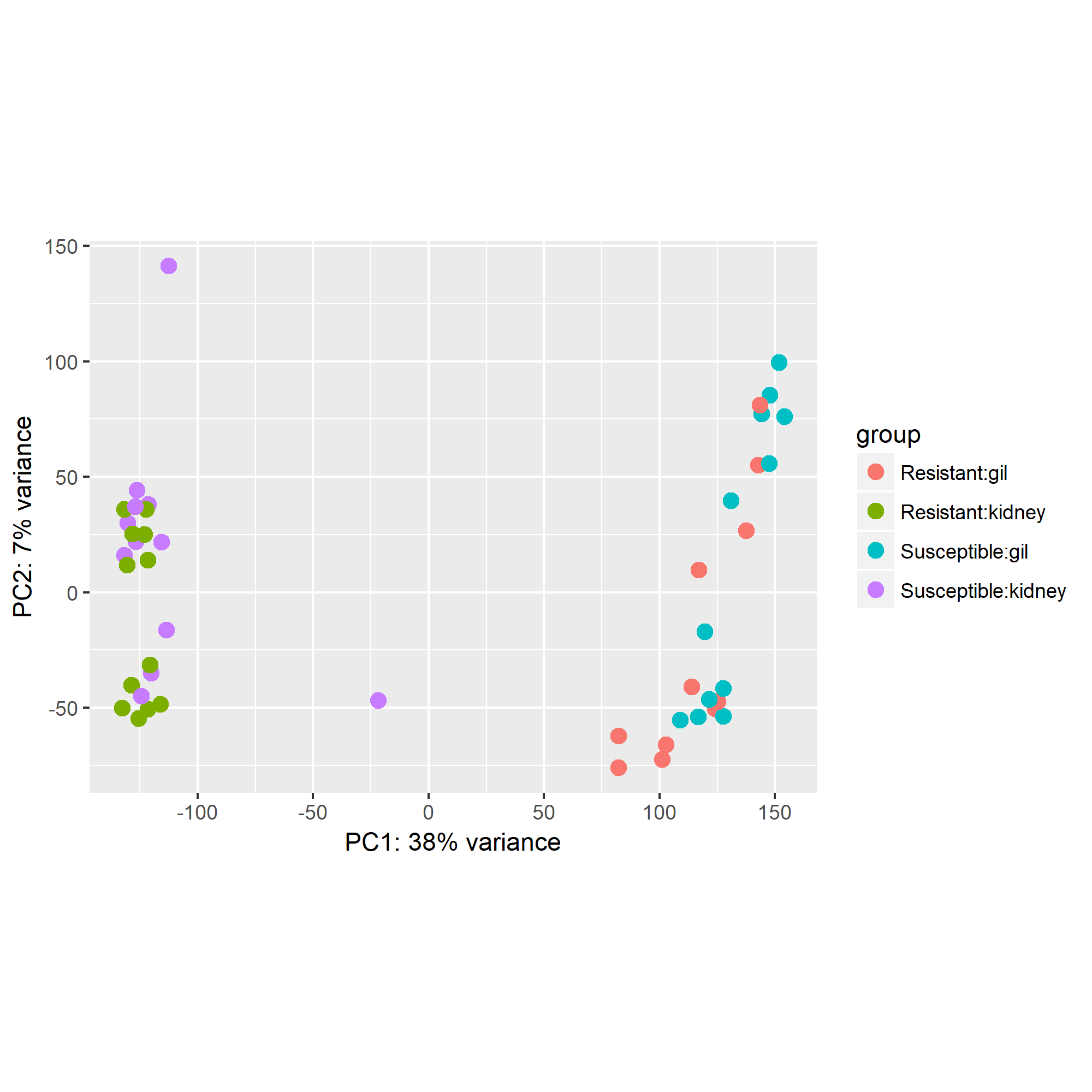

Supplement: Supplementary file 1 — Additional file 1. Principal component analysis of all RNA sequenced samples. RNA-Seq samples clustered according to their gene expression. Outliers were discarded for further analyses. [file 12864_2020_6694_MOESM1_ESM.png]
